# Phase II study of cisplatin, ifosfamide, and irinotecan with rhG-CSF support in patients with stage IIIb and IV non-small-cell lung cancer

**DOI:** 10.1038/sj.bjc.6601230

**Published:** 2003-09-09

**Authors:** A Fujita, T Ohkubo, H Hoshino, H Takabatake, S Tagaki, K Sekine, S Abe

**Affiliations:** 1 Division of Respiratory Disease, Minami-ichijo Hospital, South-1 West-13, Chuo-ku, Sapporo 060-0061, Japan; 2 Third Department of Internal Medicine, Sapporo Medical University School of Medicine, Sapporo, Japan

**Keywords:** non-small-cell lung cancer, phase II study, irinotecan, RhG-CSF

## Abstract

A phase II study of cisplatin, ifosfamide, and irinotecan with recombinant human granulocyte colony stimulating factor (rhG-CSF) support was conducted in previously untreated patients with stage IIIB or IV non-small-cell lung cancer (NSCLC). Between June 1998 and August 2001, 50 patients were registered in this phase II study. Cisplatin (20 mg m^−2^) and ifosfamide (1.5 g m^−2^) were administered on days 1–4 and irinotecan (60 mg m^−2^) was given on days 1, 8, and 15, respectively. This regimen was repeated every 4 weeks. rhG-CSF was administered subcutaneously at a dose of 50 *μ*g m^−2^ on days 5–18 except on the days of irinotecan treatment. In total, 49 patients were assessable for toxicity and response and 50 for survival. In all, 33, patients (67.3%; 95% confidence interval 57.4–77.2%) achieved an objective response. The median response duration was 192 days and the median time to progression for 49 patients was 170 days. The median survival time was 540 days with 1- and 2-year survival rates of 63.5 and 30.7%, respectively. Grade 3 or 4 neutropenia and thrombocytopenia developed in 63.3 and 38.8% of the patients, respectively. In conclusion, the combination of cisplatin, ifosfamide, and irinotecan with rhG-CSF support was highly effective for the treatment of stage IIIB or IV NSCLC with acceptable toxicities.

In the 1990s, a number of new chemotherapeutic agents such as the taxanes, vinorelbine, gemcitabine, and irinotecan were introduced into the management of advanced non-small-cell lung cancer (NSCLC). Irinotecan is a semisynthetic water-soluble derivative of camptothecin. Its mechanism of action is the inhibition of DNA topoisomerase I through the formation of topoisomerase I–DNA cleavable complexes (Hsiang *et al*, 1985; Hertzberg *et al*, 1989). A phase II study showed a high clinical efficacy of irinotecan in previously untreated NSCLC, with patients responding at a rate of 32% (Fukuoka *et al*, 1992). In a phase II study of irinotecan and cisplatin in patients with stage IIIB or IV disease, the response rate was 52% and the median survival time was 52 weeks (Masuda *et al*, 1998). In order to develop a more effective regimen, studies have been conducted in which the dose intensity of irinotecan and/or cisplatin has been increased (Masuda *et al*, 1994; Kobayashi *et al*, 1998; Ueoka *et al*, 2001), and in which various triplet-drug combination therapies using irinotecan, cisplatin, and another drug have been explored (Shinkai *et al*, 1994). Ifosfamide is an analogue of cyclophosphamide and shows significant activity against NSCLC (Costanza *et al*, 1978).

In the light of these previous studies, we conducted a phase I study of escalating dose of irinotecan combined with a fixed schedule of cisplatin and ifosfamide with recombinant human granulocyte colony stimulating factor (rhG-CSF) support in advanced NSCLC (Fujita *et al*, 1999). The combination of cisplatin and ifosfamide at respective doses of 20 mg m^−2^ and 1.5 g m^−2^ was administered on days 1–4, and irinotecan was given on days 1, 8, and 15, starting at 40 mg m^−2^ and increased in 10 mg m^−2^ increments. The dose of 60 mg m^−2^ was defined as the recommended dose of irinotecan for the subsequent phase II study. In chemotherapy-naive patients, a very promising response rate of 65.7% and a median survival time of 513 days were obtained in this trial.

Based on these findings, we performed a phase II study in order to determine the antitumour activity and toxicity of a combination of cisplatin, ifosfamide, and irinotecan with rhG-CSF support for the treatment of previously untreated patients with stage IIIB and IV NSCLC.

## PATIENTS AND METHODS

### Patient selection

Patients were enrolled in the study if they met all of the following eligibility criteria and did not meet any of the exclusion criteria: (1) histologically or cytologically confirmed advanced NSCLC (stage IIIB or IV), (2) no prior chemotherapy, (3) a life expectancy of at least 3 months, (4) a performance status (PS) of 0–1 on the ECOG scale, (5) between 15 and 75 years of age, (6) presence of bidimensionally measurable disease, (7) adequate bone marrow reserve (haemoglobin ⩾9 g dl^−1^, leucocyte count ⩾4000 mm^−3^, platelet count ⩾100 000 mm^−3^), renal function (serum creatinine <1. 5 mg dl^−1^), and liver function (AST, ALT < twice the upper limit of normal, and serum total bilirubin < 1.5 mg dl^−1^), and (8) the patient providing written informed consent for participation. The protocol was approved by an ethics review board at the Minami-ichijo Hospital.

Exclusion criteria were as follows: (1) severe concurrent medical conditions, (2) pregnant or nursing mothers, (3) active concomitant malignancy, (4) active uncontrolled infection, (5) intestinal paralysis and obstruction, (6) interstitial pneumonia or pulmonary fibrosis, and (7) large amount of ascites and/or pleural effusion.

### Evaluation

Mandatory preregistration evaluations included a baseline history, physical examination, chest X-ray, chest computed tomography (CT), bronchoscopy, head magnetic resonance imaging or CT, abdominal CT or ultrasonography, bone scintigraphy, complete blood cell counts (CBC) with differential, routine chemistry profiles, urinalysis, ECG, and pulmonary function test. During the chemotherapy courses, all patients were reviewed daily for symptoms of toxicity and underwent clinical examination. Complete blood cell count, including differential, was performed at least twice weekly. Chest X-ray, routine chemistry profiles, and urinalysis were performed at least once weekly. Tumour responses were evaluated after every course on measurable lesions determined before registration by repeating the appropriate radiographic studies. WHO evaluation criteria were used for efficacy analysis (World Health Organization, 1979). Toxicities were graded according to common toxicity criteria of the Eastern Cooperative Oncology Group (ECOG) (Oken *et al*, 1982).

### Treatment design

Ifosfamide at a dose of 1.5 g m^−2^ was diluted in 500 ml of 5% glucose solution and given over a period of 2 h as an i.v. infusion followed by an i.v. infusion of cisplatin at a dose of 20 mg m^−2^ diluted in 100 ml of 0.9% saline solution over 30 min on days 1–4. Irinotecan at a dose of 60 mg m^−2^ was dissolved in 250 ml of 0.9% saline solution and infused over a period of 90 min on days 1, 8, and 15. These dose and treatment schedules were designed on the basis of our phase I study in patients with advanced NSCLC (Fujita *et al*, 1999). rhG-CSF was administered subcutaneously at a dose of 50 g m^−2^ on days 5–18 except on the days of irinotecan treatment. All the patients received azasetron 10 mg and dexamethasone 32 mg intravenously on days 1–4 and azasetron only on days 8 and 15 before the irinotecan infusion. Mesna was given intravenously at 20% of the dose of ifosfamide at 0, 4, and 8 h after the administration of ifosfamide. Antidiarrhoeal drugs were not used prophylactically. If grade 2 or worse diarrhoea was observed, a daily dose of 7.5 g Hange-shasinto (a Kampo medicine) divided into three portions was given. Hange-shasinto is an antio-diarrhoeal agent with no inhibitory effect on intestinal motility.

The dose levels and treatment schedule were modified in order to avoid severe toxicity. Irinotecan was administered on days 8 and 15 when all of the following three conditions were met on the day of treatment: leucocyte count ⩾2000 mm^−3^, platelet count ⩾50 000 mm^−3^, and no grade 3 or worse diarrhoea. Cycles were repeated every 4 weeks if the leucocyte count was ⩾4000 mm^−3^ or the platelet count was ⩾100 000 mm^−3^. The dose of irinotecan for the subsequent course was reduced by 10 mg m^−2^ if the leucocyte count was <1000 mm^−3^, or if the platelet count was <25 000 mm^−3^, and if irinotecan was omitted on days 8 and/or 15 due to toxicity. If grade 4 diarrhoea occurred, the dose of irinotecan for the next course was reduced by 10 mg m^−2^. Dose delays of less than 1 week were permitted and the treatment was continued at the same doses after recovery from toxicity. If recovery from toxicity took more than 1 week but less than 2 weeks, the dose of irinotecan was reduced by 10 mg m^−2^. Patients were excluded from the study if recovery took longer than 2 weeks. Patients who responded to treatment could continue therapy according to the above dose modification procedure until disease progression or the development of unacceptable toxicity.

### Statistical analysis

The Kaplan–Meier method was used to estimate the response duration, time to progression, and overall survival. Time to progression and survival were calculated from the first day of chemotherapy. The primary end point of this study was the assessment of objective response rate. We assumed that a response rate of 70% would be potentially useful, while a rate of 50% would be the lower limit of interest. These response rates were based on the results of our previous phase I study in the subset of patients treated with the same dose of irinotecan and the phase II study of cisplatin and irinotecan (Masuda *et al*, 1998), respectively. Our design had a statistical power of greater than 80%, with a less than 5% error. In all, 50 patients were required in this phase II study, assuming that less than 20% of the patients entered would not be assessable for response.

## RESULTS

### Patient characteristics

Between June 1998 and August 2001, 50 patients were registered in this phase II study. One patient was withdrawn from the study on that day because of brain infarction on day 4 in the first course. Therefore, 49 patients were assessable for toxicity and response. Patient characteristics are listed in Table 1

**Table 1:** Patient characteristics

**Characteristics**	**No of patients**
*Sex*	
Male	31
Female	19
	
*Age (years)*	
Median	57
Range	38–71
	
*Performance status (ECOG)*	
0	7
1	43
*Histology*	
Adenocarcinoma	40
Squamous cell carcinoma	8
Large cell carcinoma	2
	
*Stage*	
IIIB	16
IV	34
	
*Site of metastasis*	
Lung	10
Brain	14
Bone	10
Liver	2
Adrenal	4
Abdominal LN	2
Carcinomatous pleurisy	17

. There were 31 men and 19 women with a median age of 57 years (range 38–71 years). Seven patients had a PS of 0, and 43 patients a PS of 1. Clinical stage was IIIB in 16 patients and IV in 34. Of the 34 patients with stage IV disease, the sites of metastasis included the lung in 10, brain in 14, bone in 10, liver in two, and adrenal in four. Of the17 patients with carcinomatous pleurisy, 14 had stage IV and three had stage IIIB disease. In total, 40 patients were found to have an adenocarcinoma.

### Treatment delivery

Of the 50 patients, 39 received three or more courses of chemotherapy. One patient was removed from the study because of brain infarction on day 4 of the first course. In 10 patients, chemotherapy was discontinued after two courses due to a complete abscence of efficacy. The mean number of chemotherapy courses was 4.3 in 50 patients.

In 11 patients, irinotecan treatment was omitted on days 8 and/or 15 of the first course because of toxicity. The reasons for cessation of irinotecan treatment on days 8 and/or 15 at each course are listed in Table 2

**Table 2:** Dose modification

		**Reasons for cessation of irinotecan treatment**				
**Course**	**No. of patients**	**On Day 8**		**On Day 15**		**Dose intensity**
First	50	Total	4	Total	12	90.0
		Brain infarction	1	Brain infarction	1	
		Infection	1	Pneumonitis	1	
		Thrombocytopenia	1	Diarrhoea	1	
		Diarrhoea	1	Leucopenia	6	
				Thrombocytopenia	2	
				Leucopenia and Thrombocytopenia	1	
						
Second	49	Total	0	Total	12	89.5
			1	Infection	1	
				Leucopenia	6	
				Thrombocytopenia	3	
				Leucopenia and Thrombocytopenia	1	
						
						
Third	39	Total	1	Total	19	78.5
		Leucopenia		Leucopenia	5	
				Thrombocytopenia	7	
				Leucopenia and Thrombocytopenia	7	

, which shows that the most common reasons for cessation were leucopenia and/or thrombocytopenia. At each course, the percentage of the projected irinotecan dose actually administered was 90.0% at the first course, 89.5% at the second course, and 78.5% at the third course. Only 17 patients received three or more courses of chemotherapy without dose modification.

### Response and survival

Of the 50 patients, 49 were assessable for response. No patient showed a complete response, but 33 patients achieved a partial response (PR), 14 no change, and one patient had progressive disease. The overall response rate was 67.3% (95% confidence interval (CI) 57.4–77.2%). The response rates for stage IIIB and IV were 62.5% (10 of 16 patients) and 69.7% (23 of 33 patients), respectively. The median time to remission was 52 days and the median response duration was 192 days. The median time to progression for 49 patients was 170 days. Of the 11 patients assessable for response to brain metastases, seven achieved PR. The combined treatment was also effective in 11 of 17 patients with carcinomatous pleurisy. Of 16 patients with stage IIIB disease, thoracic radiotherapy was performed on four patients in remission after three or more courses of chemotherapy. After the discontinuation of this study, 38 patients were entered in the two different phase I studies of carboplatin, docetaxel, and irinotecan with rhG-CSF support (Fujita *et al*, 2000b, 2002).

**Figure 1:**
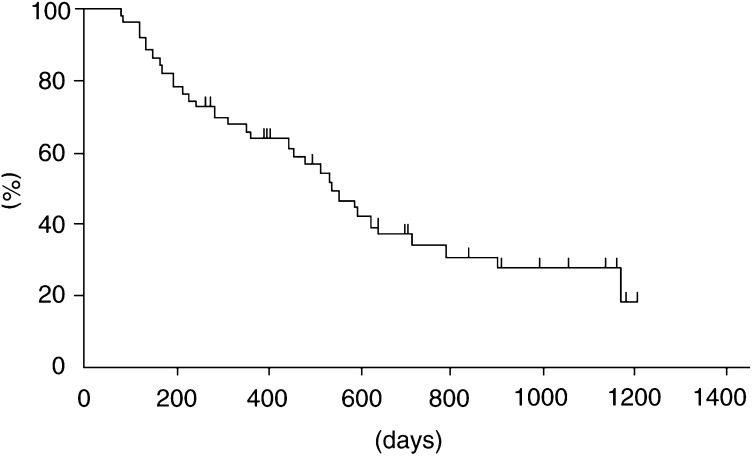
Overall survival of 50 patients.

**Figure 2:**
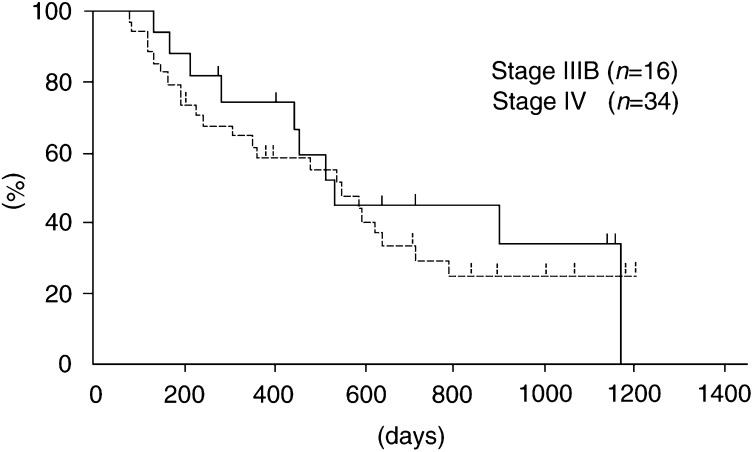
Over all survival according to clinical stage.

All patients were assessed for survival, on the basis of intent to treat. With a median follow-up of 923 days, 17 patients were still alive. The median survival time was 540 days (95% CI, 522–558 days) with 1- and 2-year survival rates of 63.5 and 30.7%, respectively (Figure 1

). According to clinical stage, the median survival times were 537 days in stage IIIB and 549 days in stage IV. The 1- and 2-year survival rates in patients with stage IIIB disease were 74.5 and 44.7%, compared with 58.4 and 28.9%, respectively, in patients with stage IV disease (Figure 2

).

### Toxicity

Toxicities occurring during the three courses are listed in Table 3

**Table 3:** Toxicity

	**CTC grade**					**⩾grade 3**	
**Toxicity**	**0**	**1**	**2**	**3**	**4**	**No of patients**	**%**
*Haematological*							
Leucopenia	2	7	12	22	6	28	57.1
Neutropenia	2	10	6	11	20	31	63.3
Thrombocytopenia	17	3	10	9	10	19	38.8
Anaemia	1	4	27	17		17	34.7
							
*Renal*							
BUN	24	22	3	0	0	0	0.0
Creatinine	49	0	0	0	0	0	0.0
Haematuria	20	29	0	0	0	0	0.0
							
*Liver*							
AST	24	22	2	1	0	1	2.0
ALT	24	21	3	1	0	1	2.0
Bilirubin	49	0	0	0	0	0	0.0
							
*Gastrointestinal*							
Nausea	7	6	9	27		27	55.1
Anorexia	7	4	9	29		29	59.2
Vomiting	17	14	18	0	0	0	0.0
Diarrhoea	39	3	4	0	3	3	6.1
Ulcer	0	0	1	0	0	0	0.0
							
*Pulmonary*							
Pneumonitis	0	0	1	0	0	0	0.0
							
*Cardiovascular*							
Cardiac-ischaemia	0	0	0	1	0	1	2.0

. The most significant toxicity was myelosuppression. Details of haematological toxicity at each course are listed in Table 4

**Table 4:** Haematological toxicity

	**First course**	**Second course**	**Third course**
*Leucopenia*			
Median nadir (× 10^3^ mm^−3^)	2.7	2.9	1.9
Range	0.3–5.5	0.6–5.1	0.5–5.1
Day nadir	15.0	15.0	15.0
			
*Neutropenia*			
Median nadir (× 10^3^ mm^−3^)	1.3	1.5	0.8
Range	0.0–3.8	0.1–3.7	0.0–3.1
Day nadir	15.0	15.0	15.0
			
*Thrombocytopenia*			
Median nadir (× 10^3^ mm^−3^)	12.6	10.9	6.4
Range	2.9–27.0	2.5–21.3	0.9–19.9
Day nadir	15.0	15.0	15.0
No. of patients transfused	1	0	7
			
*Anaemia*			
Median nadir (g dl^−1^)	10.0	9.1	8.3
Range	7.7–12.5	7.3–11.6	7.0–11.1
No. of patients transfused	5	10	20

. Grade 3 and 4 leucopenia occurred in 22 and six patients, respectively. The leucocyte nadir occurred at around day 15 during each of the three courses. Grade 4 neutropenia developed in 20 patients, but it never lasted for more than 5 days except in one patient. Eight patients experienced neutropenic fever, but no patients suffered from neutropenic sepsis. Nine and 10 patients experienced grade 3 and 4 thrombocytopenia, respectively. Eight patients required platelet transfusion. Anaemia was prominent and 26 patients required red blood cell transfusion. Increase in the number of the chemotherapy course was associated with severity of myelotoxicity. Nausea and anorexia of grade 2 or worse were observed in most patients. Diarrhoea worse than grade 2 was observed in three patients, but the symptoms proved to be transient. In all, 25 patients showed elevation of serum transaminase, although this condition was transient and not significantly severe. Pneumonitis developed in one patient and was improved with corticosteroid treatment. There were no treatment-related deaths.

## DISCUSSION

As a single agent, irinotecan results in a significant response rate in previously untreated NSCLC patients, and with the combination of irinotecan and cisplatin, an encouraging response rate of 52% has been found (Masuda *et al*, 1998). In the two Japanese multi-institutional randomised trials (Masuda *et al*, 1999; Niho *et al*, 1999), cisplatin plus irinotecan significantly improved survival compared to cisplatin plus vindesine in patients with metastatic NSCLC, as determined by multivariate analysis (Fukuoka *et al*, 2000). The present study was designed to test whether the combination of cisplatin and irinotecan with ifosfamide can develop a more effective regimen. With prophylactic administration of rhG-CSF, from May 1994 to June 1995, we conducted a phase I study of irinotecan on days 1, 8, and 15 combined with a fixed schedule of cisplatin and ifosfamide and determined the recommended dose of irinotecan as 60 mg m^−2^ for the subsequent phase II study (Fujita *et al*, 1999).

The current phase II study demonstrates an encouraging response rate of 67.3% comparable to the previously conducted phase I study. There was no significant difference in the response rate according to clinical stage. The response rate was also high in patients with cerebral metastasis (Fujita *et al*, 2000a) and findings such as disappearance of pleural effusion, negative cytology, and improvement of pleural thickening were obtained in 11 of 17 patients with carcinomatous pleurisy (Fujita *et al*, 2001).

The median survival time and 1- and 2-year survival rates in all patients were 540 days, 63.5 and 30.7%, respectively, and these results were considered satisfactory. According to clinical stage, the 1- and 2-year survival rates in patients with stage IIIB disease were 74.5 and 44.7%, compared with 58.4 and 28.9%, respectively, in patients with stage IV disease. The survival time obtained in the current study is in the upper range of reported trials of combination chemotherapy for NSCLC. However, due to patient's selection, it is difficult to reach valid conclusions regarding the most effective chemotherapy based only on the historical results.

Recently, new drugs have demonstrated satisfactory activity as second-line treatment in patients with NSCLC. After discontinuation in the present study, 38 patients were registered into the two different phase I studies of carboplatin, docetaxel, and irinotecan with rhG-CSF support (Fujita *et al*, 2000b; 2002). Both these phase I studies demonstrated an encouraging response rate in patients with prior chemotherapy. The effect of such a second-line chemotherapy on the results of this trial is unclear. However, the role of a second-line chemotherapy should be taken into consideration.

Grade 3 or 4 neutropenia developed in 63.3% of the patients in the present trial. The severity of neutropenia was not completely eliminated by rhG-CSF support. However, grade 4 neutropenia lasting more than 5 days occurred in only one patient. The availability of rhG-CSF apparently made it possible to reduce the duration of neutropenia. Grade 3 or 4 thrombocytopenia occurred in 38.8% of the patients in this trial. Only 17 patients received three or more courses of chemotherapy without dose modification. Haematological toxicity was severe despite the dose modification. Three patients experienced grade 4 diarrhoea, were treated with Hange-shasinto, and showed improvement.

In conclusion, the combination of cisplatin, ifosfamide, and irinotecan with rhG-CSF support was highly effective for the treatment of stage IIIB or IV NSCLC. Haematological toxicity was prominent but manageable. Phase III trials are required to determine whether this combination of three drugs with rhG-CSF support will improve the response rate and survival in previously untreated NSCLC patients.
